# Mendelian randomization study of the relationship between blood and urine biomarkers and lung cancer

**DOI:** 10.3389/fonc.2024.1453246

**Published:** 2024-12-02

**Authors:** Haihua Huang, Haijun Zheng

**Affiliations:** The First People's Hospital of Chenzhou, Chenzhou, China

**Keywords:** lung cancer, biochemical markers in blood and urine, Mendelian randomization, scRNA-seq, scPagwas method

## Abstract

**Introduction:**

Identifying suitable biomarkers is crucial for exploring the pathogenesis, early screening, and therapeutic monitoring of lung cancer. This study aims to analyze comprehensively the associations between lung cancer and biomarkers in blood and urine.

**Methods:**

Bidirectional two-sample Mendelian randomization (MR) was used to evaluate the potential causal relationships between blood and urine biomarkers and lung cancer. We obtained Single nucleotide polymorphisms (SNPs) related to lung cancer from the 2021 Finnish database of genome-wide association studies, including small cell lung cancer (SCLC), total non-small cell lung cancer (NSCLC), lung adenocarcinoma (LAC), and lung squamous cell carcinoma (LSCC).Data on blood and urine biomarkers were derived from the UK Biobank cohort, comprising 376,807 participants.

**Results:**

We found a potential inverse causal relationship between total bilirubin and SCLC (β=-0.285, P=0.015, FDR=0.12). Urate was inversely associated with NSCLC (β=-0.158, P=0.004, FDR=0.036*). Serum calcium showed a possible inverse relationship with lung squamous cell carcinoma (β=-0.256, P=0.046, FDR=0.138), while urinary creatinine was positively associated (β=1.233, P=0.024, FDR=0.216). Non-albumin proteins (β=-0.272, P=0.020, FDR=0.180) and total protein (β=-0.402, P=0.009, FDR=0.072) were inversely related to lung squamous cell carcinoma. The AST/ALT ratio was positively associated with lung adenocarcinoma (β=0.293, P=0.009, FDR=0.072). Our reverse Mendelian randomization study found a positive causal association between small cell lung cancer and serum creatinine (β=0.022, P=0.002, FDR=0.018*), while it was inversely associated with the estimated glomerular filtration rate(eGFR)(β=-0.022, P=0.003, FDR=0.027*). A positive causal relationship was also observed with cystatin C (β=0.026, P=0.005, FDR=0.045*) and glycated hemoglobin HbA1c (β=0.013, P=0.014, FDR=0.028*). A negative causal relationship was observed with Gamma_glutamyltransferase (β=-0.013, P=0.019, FDR=0.152). For non-small cell lung cancer, a negative causal relationship was found with albumin (β=-0.024, P=0.002, FDR=0.016*), while a potentially positive causal relationship was observed with cystatin C (β=0.022, P=0.006, FDR=0.054). Possible negative causal relationships were also observed with phosphate (β=-0.013, P=0.008, FDR=0.072) and urinary potassium (β=-0.011, P=0.012, FDR=0.108), while a potential positive causal relationship was observed with C-reactive protein (β=0.013, P=0.040, FDR=0.280).Regarding lung squamous cell carcinoma, an inverse causal relationship was found with eGFR (β=-0.022, P=9.58e-06, FDR=8.62×10-5*), while a positive causal relationship was observed with serum creatinine (β=0.021, P=1.16e−4, FDR=1.05×10-3*). Potential positive causal relationships were observed with Urate (β=0.012, P=0.020, FDR=0.180), urea (β=0.010, P=0.046, FDR=0.141), and glycated hemoglobin HbA1c (β=0.020, P=0.049, FDR P=0.098), whereas a potential negative causal relationship was observed with sex hormone-binding globulin(SHBG) (β=-0.020, P=0.036, FDR=0.108).Lastly, adenocarcinoma was found to have a positive causal association with alkaline phosphatase (β=0.015, P=0.006, FDR=0.033*).

**Conclusion:**

Our study provides a robust theoretical basis for the early screening and therapeutic monitoring of lung cancer and contributes to understanding the pathogenesis of the disease.

## Introduction

Lung cancer is the leading cause of cancer-related mortality (1), with limited treatment options due to most patients being diagnosed at a late stage (2). Whilst smoking is undeniably the primary global risk factor for lung cancer, environmental exposures (3), genetic factors (4), and multi-omics biomarkers (5) also drive its initiation and progression. To enhance early detection of lung cancer, high-risk individuals can undergo low-dose computed tomography (CT) screening; however, this method is plagued by high false-positive rates and patient radiation exposure, and current screening programs primarily target heavy smokers and the elderly. Although all types of lung cancer are associated with smoking, small cell lung cancer (SCLC) and squamous cell carcinoma have a higher incidence in smokers. Conversely, in never-smokers, adenocarcinomas are more prevalent, representing a larger proportion of all lung cancer cases and becoming increasingly common in younger patients, particularly never-smokers (6). To address these limitations, the use of biomarkers as potential supplements or alternatives to low-dose CT has been proposed, prompting extensive research in this area. However, current data on their clinical efficacy and their comparison with existing lung cancer screening strategies are relatively scarce. Identifying these biomarkers necessitates a deeper understanding of how tumors initiate and progress, and of the importance of the role these molecules play in this process (7).

Peripheral blood and urinary biomarkers are frequently used for diagnosing and assessing chronic disease status (8). Biochemical markers in peripheral blood and urine have been found to be abnormal in many patients with lung cancer, making them promising alternatives for lung cancer detection, although their application in clinical practice remains limited.

Mendelian randomization (MR) is a statistical method that uses genetic variations as instrumental variables (IVs) (9) to infer causal relationships between exposures and outcomes. MR integrates summary data from genome-wide association studies (GWAS), akin to a natural randomized controlled trial. Given that genotype allocation from parents to offspring is random, MR studies are less susceptible to confounding factors and reverse causation compared to traditional observational studies (10). MR has emerged as a powerful tool for identifying causal relationships between risk factors and diseases and is widely used in epidemiological research to explore potential causal associations between two traits (11).

**Figure 1:**
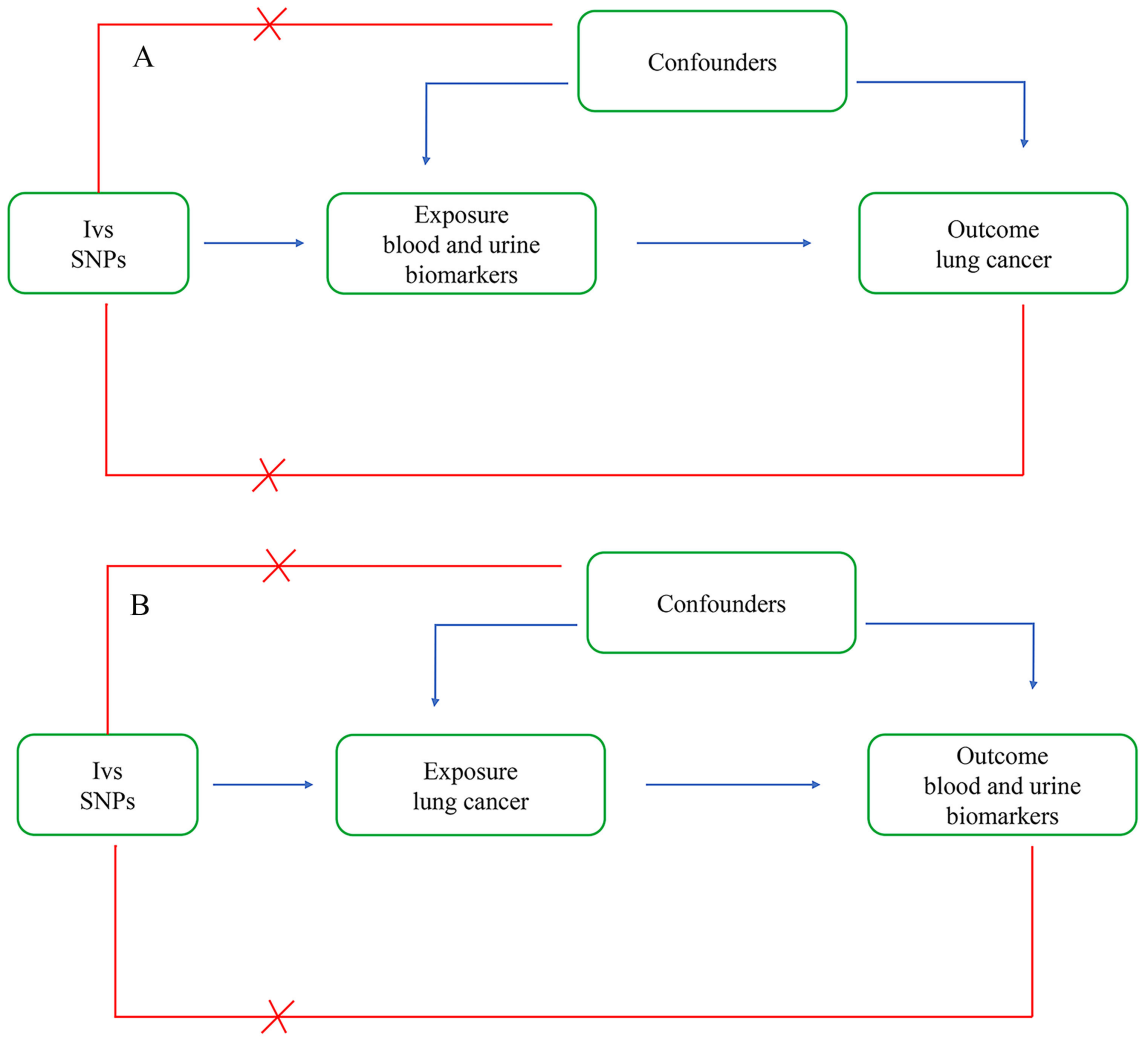
Bi-directional two-sample Mendelian randomization analysis. **(A)** Forward analysis: The exposure is blood and urine biomarkers, and the outcome is lung cancer. **(B)** Reverse analysis: The exposure is lung cancer, and the outcome is blood and urine biomarkers.

In this study, we comprehensively analyzed the associations between blood and urinary biomarkers and lung cancer. We conducted bidirectional two-sample Mendelian randomization analyses to validate the causal relationships between biomarkers and lung cancer (**Figure 1**).

## Methods

### UKB cohort serum and urine biomarker GWAS dataset

The blood and urine biomarker data used in this study were derived from the UK Biobank (UKB), which conducted a large prospective cohort study from 2006 to 2010 (12). The UKB performed laboratory tests on common biomarkers in serum (category 100080) and urine (category 100083) in a cohort with extensive phenotype and whole-genome genotype data (12). Participants’ health-related records, including age and sex, were collected via touchscreen questionnaires or verbal interviews at assessment centers. These data are publicly accessible at https://gwas.mrcieu.ac.uk/.

### Lung Cancer GWAS Dataset

The GWAS summary statistics data for lung cancer were sourced from a 2021 study in the Finnish database R10. This included data for four types: small-cell lung cancer, non-small cell lung cancer, adenocarcinoma, and squamous cell carcinoma. Small-cell lung cancer included 717 cases and 314,193 controls, non-small cell lung cancer included 5,315 cases and 314,193 controls, squamous cell carcinomas included 1,510 cases and 314,193 controls, and adenocarcinomas included 1,590 cases and 314,193 controls.

### Bidirectional two-sample Mendelian randomization data analysis

The traits investigated in this study comprised 35 blood and urine biomarkers, specifically Alanine aminotransferase, Albumin, Alkaline phosphatase, Apolipoprotein A, Apolipoprotein B, Aspartate aminotransferase, AST to ALT ratio, C-reactive protein, Calcium, Cholesterol, Creatinine, Creatinine in urine, Cystatin C, Direct bilirubin, eGFR, Gamma glutamyltransferase, Glucose, HbA1c, HDL cholesterol, IGF-1, LDL cholesterol, Lipoprotein A, Microalbumin in urine, Non-albumin protein, Phosphate, Potassium in urine, SHBG, Sodium in urine, Testosterone, Total bilirubin, Total protein, Triglycerides, Urate, Urea, Vitamin D. These included 8 liver-related, 7 cardiovascular-related, 9 kidney-related, 3 osteoarthritis-related, 2 diabetes-related, and 3 hormone-related indicators (**Supplementary Table S1**). Lung cancer types included small-cell lung cancer, non-small cell lung cancer, lung adenocarcinoma, and lung squamous cell carcinoma. The number of genome-wide significant independent loci for each trait was represented by SNPs (n), with a screening p-value of 5e-8. We ensured the independence of each SNP by setting a linkage disequilibrium (LD) threshold of r^2 < 0.001 and a clumping distance of 10,000 kb, based on the European 1000 Genomes Project reference panel (13).

We also harmonized the biomarker and lung cancer data for subsequent MR analysis. If exposure-related SNPs were missing in the outcome GWAS, we selected proxy SNPs with r^2 > 0.80. We then removed palindromic SNPs with A/T or G/C alleles to ensure consistent allelic effects of SNPs on exposure and outcome.

### Statistical methods

We carried out MR analysis to assess the causal effects of biomarkers on lung cancer, using the inverse variance weighted (IVW) method as our primary analytical approach (14). We also applied MR-Egger regression, weighted median method, and weighted and simple modes to further verify the robustness of the MR analysis results. Significant results (p < 0.05) generated by the IVW method were considered positive outcomes even if there was no significance exhibited by the other methods, provided that the direction of the beta values was consistent across the methods. To consider multiple testing, we employed a modified version of the Benjamini and Hochberg false discovery rate (FDR) procedure, tailored to the hierarchical and interdependent nature of our data (15). At each category level, we set an FDR corrected significance threshold of 0.05, based on the effective number of independent tests at each category level. With liver category p = 0.05/8 = 6.3 × 10^-3, cardiovascular p = 0.05/16 = 7.1 × 10^-3, kidney p = 0.05/9 = 5.6 × 10^-3, osteoarthritis p = 0.05/8 = 1.67 × 10^-2, diabetes p = 0.05/2 = 2.5 × 10^-2, and hormones p = 0.05/3 = 1.67 × 10^-2. For identifying more precise causal associations, we employed an FDR significance threshold of p < 0.05. We utilized the MR-Egger regression intercept to detect potential pleiotropy (16). If the MR-Egger intercept was not statistically significant (p > 0.05), there was no evidence suggestive of pleiotropy. We performed Cochran’s Q statistical analysis in the IVW mode to check for potential heterogeneity amongst the selected IVs (17). If heterogeneity was present (p < 0.05), we further validated through the IVW random effects model and IVW multiplicative random effects model. Additionally, we employed leave-one-out sensitivity analyses to test the potential influence of individual SNPs on the observed causal effects. Moreover, we assessed the strength of the IVs chosen in our study by calculating the F-statistic, with the final SNPs included in the analysis being F > 10. This enabled us to rule out the possibility of weak instrument bias affecting our estimation of the causal relationship. The formula for the F-statistic was F= R^2/(1-R^2) * (n-k-1)/k, where R^2 represented the proportion of variance explained by SNPs, n was the sample size, and k was the number of IVs included. R^2 was estimated using the MAF and β values, with the formula: R^2 = 2 * MAF * (1 - MAF) * β^2. Finally, we conducted reverse MR analysis to explore the causal effects of lung cancer on biomarkers, following the same protocol as the previous MR. All statistical analyses were conducted using R software (Version 4.3.0), with the R packages TwosampleMR and MR-PRESSO.

## Results

### Causal effects of biomarkers on lung cancer

**Figure 2:**
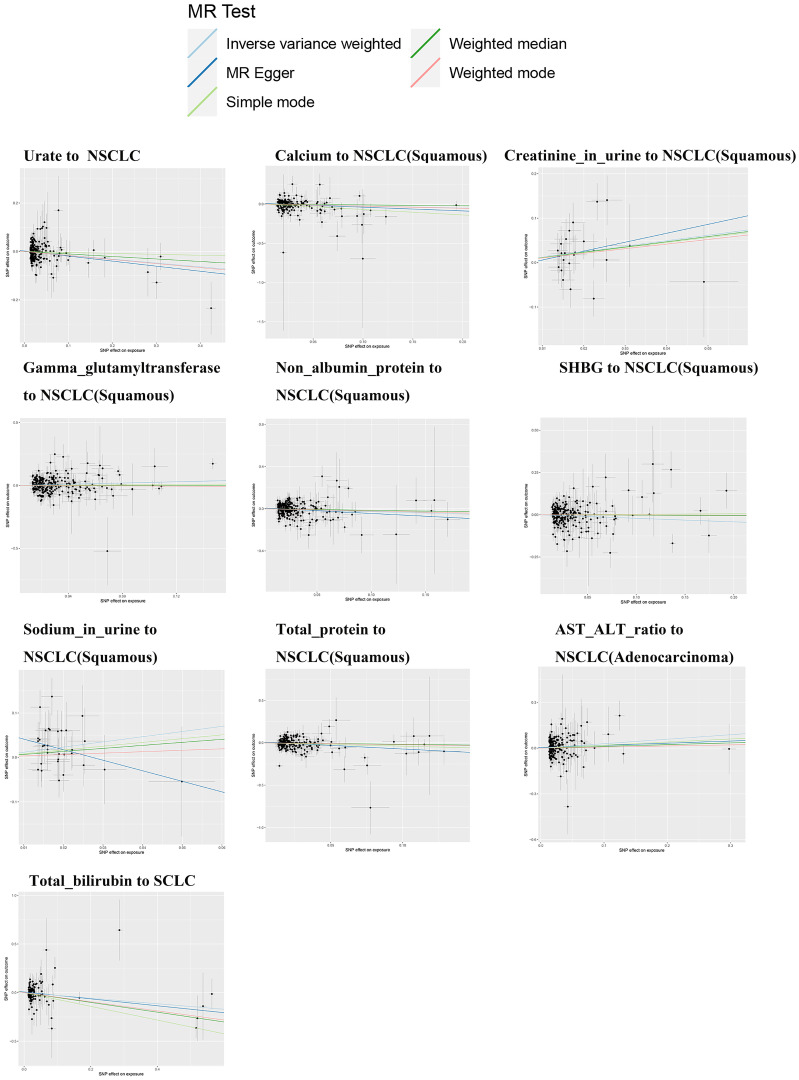
Mendelian randomization scatter plot of biomarkers and lung cancer.

The IVW analysis results shown in **Table 1** suggest a potential causal relationship between genetically predicted total bilirubin levels and a lower risk of small cell lung cancer (β: -0.285, P: 0.015, FDR: 0.12). A more precise negative causal relationship was found between urate levels and non-small cell lung cancer (β: -0.158, P: 0.004, FDR: 0.036*). Serum calcium showed a potential negative causal relationship with squamous cell carcinoma (β: -0.256, P: 0.046, FDR: 0.138), while urine creatinine showed a potential positive causal relationship with squamous cell carcinoma (β: 1.233, P: 0.024, FDR: 0.216). Gamma-glutamyltransferase also showed a potential positive causal relationship with squamous cell carcinoma (β: 0.241, P: 0.009, FDR: 0.072), while non-albumin exhibited a potential negative causal relationship with squamous cell carcinoma (β: -0.272, P: 0.020, FDR: 0.180). SHGB was potentially negatively causally related to lung squamous cell carcinoma (β: -0.209, P: 0.033, FDR: 0.297), while sodium in urine showed a potential positive causal relationship with lung squamous cell carcinoma (β: 1.166, P: 0.010, FDR: 0.090). Total protein demonstrated a potential negative causal relationship with lung squamous cell carcinoma (β: -0.402, P: 0.009, FDR: 0.072), while AST/ALT revealed a potential positive causal relationship with lung adenocarcinoma (β: 0.293, P: 0.009, FDR: 0.072). Except for the causal relationships between Gamma_glutamyltransferase, SHBG, Sodium_in_urine and lung squamous cell carcinoma, the other seven causal relationships were all validated by five types of MR analysis, and generated consistent effect estimation directions (**Supplementary Table S2**). **Figure 2** illustrates the scatter plots of the study results. After FDR correction, only the IVW estimate for urate (OR = 0.854, 95%CI = 0.766–0.952, FDR = 0.036*) remained significantly associated with small cell lung cancer.

**Table 1:** Mendelian randomization study results of biomarkers and lung cancer.

Biomarkers	Lung cancer	β	P	FDR	OR	OR (95% Cl)	
Total Bilirubin	Small cell lung cancer	-0.285	0.015	0.12	0.752	0.597	0.947
Urate	Non-small cell cancer	-0.158	0.004	0.036*	0.854	0.766	0.952
Calcium	Lung squamous cell carcinoma	-0.256	0.046	0.138	0.774	0.601	0.995
Urinary Creatinine	Lung squamous cell carcinoma	1.233	0.024	0.216	3.433	1.181	9.983
Gamma-Glutamyltransferase	Lung squamous cell carcinoma	0.241	0.009	0.072	1.272	1.060	1.526
Non-Albumin	Lung squamous cell carcinoma	-0.272	0.020	0.180	0.762	0.606	0.958
SHBG	Lung squamous cell carcinoma	-0.209	0.033	0.099	0.811	0.669	0.984
Urinary Sodium	Lung squamous cell carcinoma	1.166	0.010	0.090	3.210	1.317	7.826
Total Protein	Lung squamous cell carcinoma	-0.402	0.009	0.072	0.669	0.495	0.903
AST/ALT Ratio	Lung Adenocarcinoma	0.293	0.009	0.072	1.341	1.029	1.748

*FDR:P<0.05.

### Causal effects of lung cancer on biomarkers

Reverse MR analysis revealed causal associations of lung cancer on biomarkers. As shown in **Table 2**, IVW analysis results show that genetically predicted risk of small cell lung cancer has a positive causal relationship with Creatinine (β: 0.022, P: 0.002, FDR: 0.018*), Cystatin C (β: 0.026, P: 0.005*, FDR: 0.045*), and Haemoglobin HbA1c (β: 0.013, P: 0.014, FDR: 0.028*), a potential positive causal relationship with AST-ALT_ratio (β: 0.021, P: 0.022, FDR: 0.176), and a clear negative causal relationship with eGFR (β: -0.022, P: 0.003, FDR: 0.027*), and a potential negative causal relationship with Gamma Glutamyltransferase (β: -0.013, P: 0.019, FDR: 0.152). Non-small cell lung cancer exhibits potential positive causal relationships with Cystatin C (β: 0.022, P: 0.006, FDR: 0.054), and C-reactive protein (β: 0.013, P: 0.04, FDR: 0.28), a clear negative causal relationship with albumin (β: -0.024, P: 0.002, FDR: 0.016*), and potential negative causal relationships with phosphate (β: -0.013, P: 0.008, FDR: 0.072), and urine potassium (β: -0.011, P: 0.012, FDR: 0.108). Lung squamous cell carcinoma has a clear causal relationship with creatinine (β: 0.021, P: 1.16×10-5, FDR: 1.05×10-3*), Cystatin C (β: 0.028, P: 0.004, FDR: 0.036*), potential positive causal relationships with urate (β: 0.012, P: 0.020, FDR: 0.180), urea (β: 0.010, P: 0.046, FDR: 0.141), and Glycated Haemoglobin HbA1c (β: 0.019, P: 0.049, FDR: 0.098), and a clear negative causal relationship with eGFR (β: -0.022, P: 9.58×10-6*, FDR: 8.62×10-5*), and a potential negative causal relationship with SHBG (β: -0.020, P: 0.036, FDR: 0.108). Adenocarcinoma has a clear positive causal relationship with alkaline phosphatase (β: 0.015, P: 0.011, FDR: 0.033*).

**Table 2:** Lung cancer and biomarkers Mendelian randomization study results.

Lung cancer	Biomarker	β	P	FDR	OR	OR (95% CI)	
Small cell lung cancer	Creatinine	0.022	0.002	0.018*	1.023	1.008	1.037
	eGFR	-0.022	0.003	0.027*	0.978	0.964	0.992
	Cystatin C	0.026	0.005	0.045*	1.026	1.008	1.045
	Glycated Haemoglobin HbA1c	0.013	0.014	0.028*	1.014	1.003	1.025
	Gamma Glutamyltransferase	-0.013	0.019	0.152	0.987	0.977	0.998
	AST/ALT	0.021	0.022	0.176	1.021	1.003	1.039
Non-small cell lung cancer	Albumin	-0.024	0.002	0.016*	0.977	0.963	0.992
	Cystatin C	0.022	0.006	0.054	1.023	1.006	1.039
	Phosphate	-0.013	0.008	0.072	0.987	0.978	0.997
	Urinary Potassium	-0.011	0.012	0.108	0.990	0.981	0.998
	C Reactive Protein	0.013	0.04	0.28	0.025	1.013	1.001
Lung squamous cell carcinoma	eGFR	-0.022	9.58×10-6	8.62×10-5*	0.979	0.969	0.988
	Creatinine	0.021	1.16×10-5	1.05×10-3*	1.021	1.010	1.031
	Cystatin C	0.028	0.004	0.036*	1.029	1.009	1.049
	Urate	0.012	0.020	0.180*	1.012	1.002	1.022
	SHBG	-0.020	0.036	0.108	0.981	0.963	0.999
	Urea	0.010	0.046	0.141	1.010	1.000	1.019
	Glycated Haemoglobin HbA1c	0.019	0.049	0.098	1.019	1.000	1.038
Lung adenocarcinoma	Alkaline Phosphatase	0.015	0.011	0.033*	1.015	1.003	1.027

*FDR:P<0.05.

**Figure 3:**
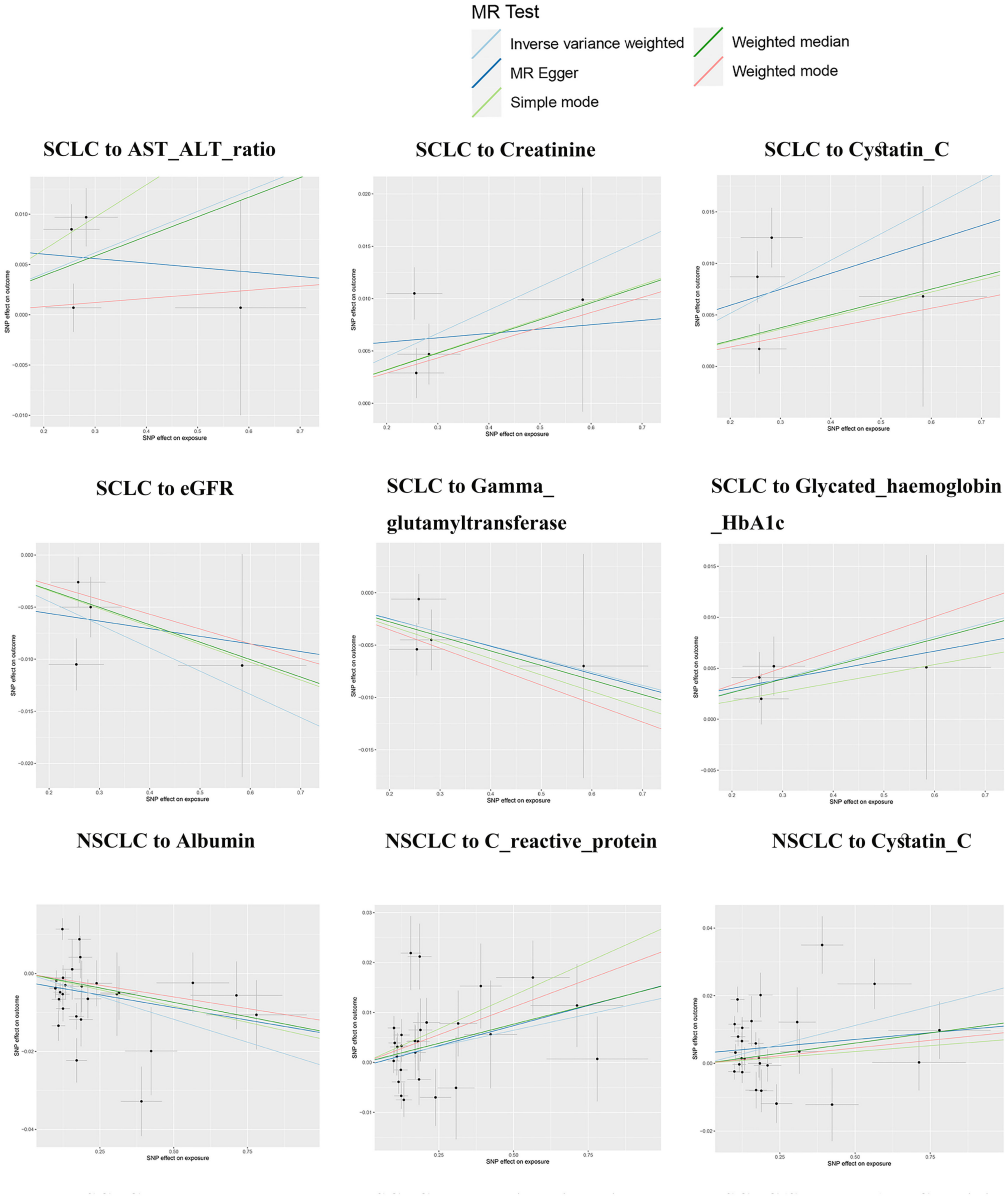
Lung cancer and biomarkers Mendelian randomization scatter plot.

**Figure 4:**
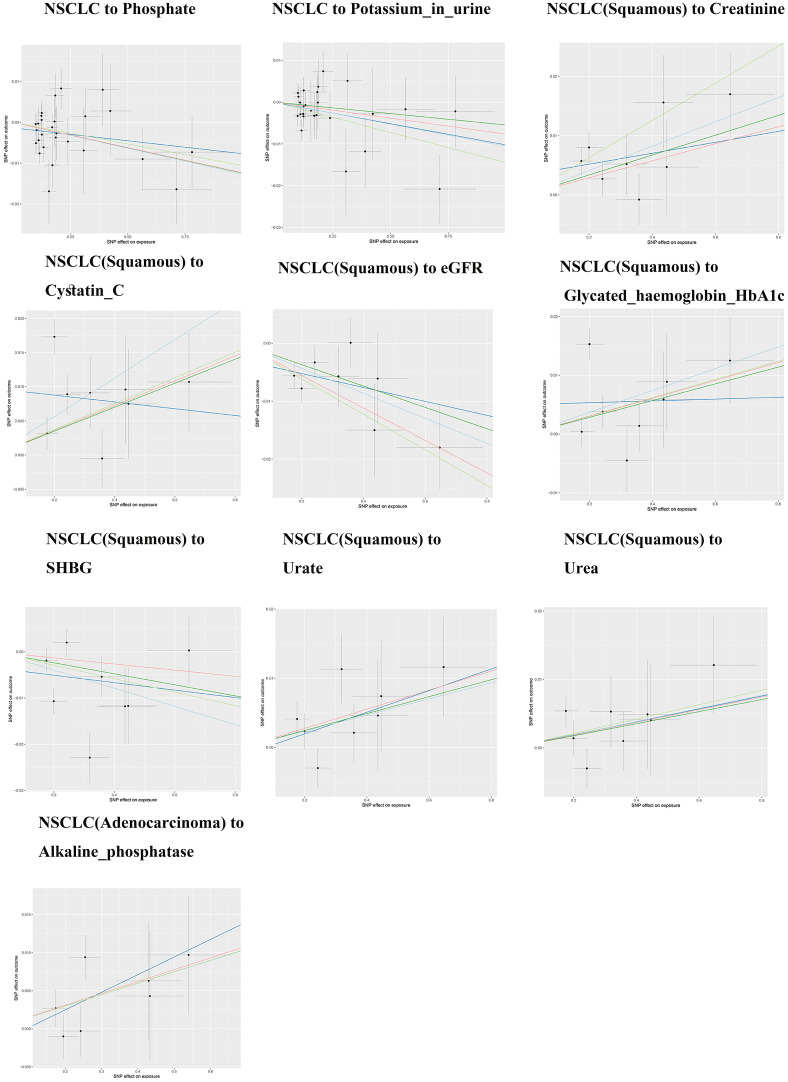
Lung cancer and biomarkers Mendelian randomization scatter plot.

Except for small cell lung cancer with AST/ALT ratio and squamous cell carcinoma with Cystatin_C, the other 17 causal associations were all validated by all five types of MR analysis (**Supplementary Table S3**). The scatter plots of each test are shown in **Figures 3** and **4**. After FDR correction, associations remained significant between small cell lung cancer and creatinine, eGFR, cystatin C, glycated haemoglobin A1c, non-small cell lung cancer and albumin, lung squamous cell carcinoma and eGFR, creatinine, cystatin C. Lung adenocarcinoma and alkaline phosphatase.

### Sensitivity analysis

To further validate the causal associations, sensitivity analyses were performed to assess pleiotropy and heterogeneity in the MR results. The MR Egger intercept test showed no significant evidence of pleiotropy (all P-values > 0.05) (**Supplementary Table S4**). However, evidence of heterogeneity was found in some cases based on Cochran’s Q test (**Supplementary Table S5**). For results exhibiting heterogeneity, we re-evaluated them using the IVW random-effects model and the multiplicative random-effects model. The results remained consistent with those obtained from the IVW method (**Supplementary Tables S6, S7**). Additionally, leave-one-out analyses indicated that no single SNP was driving the identified causal associations (**Supplementary Figures S1, S2**).

## Discussion

In this study, we initially employed bidirectional Mendelian randomization (MR) analyses to investigate the causal relationships between 35 blood and urine biomarkers and various types of lung cancer. In the forward MR analysis, we identified seven potential causal associations. Five biomarkers showed inverse causal relationships with lung cancer risk: total bilirubin with small cell lung cancer (SCLC), urate with non-small cell lung cancer (NSCLC), serum calcium with lung squamous cell carcinoma (LSCC), non-albumin proteins with LSCC, and total protein with LSCC. Conversely, two biomarkers exhibited positive causal relationships with lung cancer phenotypes: urinary creatinine with LSCC and the AST/ALT ratio with lung adenocarcinoma (LADC).

In the reverse MR analysis, we identified 17 potential causal relationships. SCLC showed positive causal relationships with creatinine, cystatin C, and glycated hemoglobin A1c (HbA1c), and negative causal relationships with eGFR and gamma-glutamyl transferase (GGT). NSCLC showed positive causal relationships with cystatin C and C-reactive protein (CRP), and negative causal relationships with albumin, phosphate, and urinary potassium. LSCC showed positive causal relationships with creatinine, urate urea, and HbA1c, and negative causal relationships with eGFR and SHBG. LADC showed a positive causal relationship with alkaline phosphatase (ALP).

After adjusting for the false discovery rate (FDR), we confirmed 10 more robust causal associations (**Table 3**), including: urate as a protective factor for NSCLC; SCLC increasing blood creatinine levels, decreasing eGFR, increasing cystatin C levels, and increasing HbA1c levels; NSCLC decreasing albumin levels; LSCC decreasing eGFR and increasing blood creatinine cystatin C levels; and LADC increasing ALP levels. Fourteen other potential causal relationships were identified.

**Table 3:** Results of bidirectional Mendelian randomization study on lung cancer and biomarkers (FDR < 0.05).

Biomarkers/lung cancer	Biomarkers/Lung cancer	β	P	FDR	OR	OR (95% CI)	
Urate	Non-Small Cell Lung Cancer	-0.158	0.004*	0.036*	0.854	0.766	0.952
Small cell lung cancer	Creatinine	0.022	0.002*	0.018*	1.023	1.008	1.037
	eGFR	-0.022	0.003*	0.027*	0.978	0.964	0.992
	Cystatin C	0.026	0.005*	0.045*	1.026	1.008	1.045
	Glycated Haemoglobin HbA1c	0.013	0.014*	0.028*	1.014	1.003	1.025
Non-small cell lung cancer	Albumin	-0.024	0.002*	0.016*	0.977	0.963	0.992
Lung squamous cell carcinoma	eGFR	-0.022	9.58×10-6*	8.62×10-5*	0.979	0.969	0.988
	Creatinine	0.021	1.16×10-5*	1.05×10-3*	1.021	1.01	1.031
	Cystatin C	0.028	0.004*	0.036*	1.029	1.009	1.049
Lung adenocarcinoma	Alkaline Phosphatase	0.015	0.011*	0.033*	1.015	1.003	1.027

*P<0.05.

Bilirubin possesses potent antioxidative properties, which may help protect respiratory tissues from oxidative stress (18–21). Maria J. Monroy-Iglesias et al. (22) found in a Cohort Study and Meta-Analysis that total bilirubin is a protective factor for lung cancer. Laura Jane Horsfall et al. (23) also demonstrated via Mendelian randomization study using total bilirubin single nucleotide polymorphisms (SNPs) that total bilirubin is a protective factor for lung cancer, particularly among heavy smokers. Our study results corroborate these findings, confirming that plasma total bilirubin levels serve as a protective factor for SCLC.

Serum urate exhibits potent antioxidant properties *in vitro* and is the most abundant antioxidant molecule in human blood (24, 25). It’s estimated that up to 50% of antioxidant capacity in human blood is attributable to the action of serum urate (26). Not only is urate highly concentrated in the blood, but it’s also present in high amounts in human respiratory tissues and epithelial lining fluid of the airways, potentially providing an important first line of defense against environmental oxidants in smoke and pollutants (27, 28). While Haruka Fujikawa et al. (29) suggested a certain negative correlation between urate and lung cancer, Laura J. Horsfall et al. (30) did not find an association in their cohort and one-sample Mendelian randomization study. However, an observational study by A. Bozkır (31) found that lung cancer patients had significantly higher urate levels than healthy controls. Our study results show that urate has a protective effect on NSCLC, while LSCC increases the level of serum urate.

Studies have found that signals from the 1,25(OH)2D3 receptor (VDR) and calcium-sensing receptor (CaSR) can inhibit tumor proliferation and metastasis, and promote tumor differentiation and apoptosis (32, 33). However, Yumie Takata et al. (34) found no correlation between calcium intake and lung cancer, while Haihao Yan et al. (35) showed via a Mendelian randomization study that serum calcium is a protective factor for lung cancer, including SCLC, LADC, and LSCC. Our study supports the latter finding, showing that serum calcium is a protective factor for LSCC.

The term “non-albumin” refers to proteins other than albumin in the blood, the majority of which are immunoglobulins. Extensive research has been conducted on the protective role of immunoglobulins against tumors. Our results align with these findings, showing an inverse relationship between non-albumin proteins and LSCC.

Plasma total protein represents the sum of all proteins in the blood, primarily including albumin and globulin. These proteins play crucial roles in maintaining fluid balance, transporting nutrients, immune responses, and blood clotting processes. The level of plasma total protein can reflect the body’s nutritional status, liver function, kidney function, and immune status. Füsun Sahin (36) found no difference in total protein levels between lung cancer patients and healthy individuals in a cross-sectional study, whereas Priyanka Gaur (37)reported significantly lower total protein levels in lung cancer patients compared to healthy controls. Our study indicates that plasma total protein is a protective factor for LSCC.

Creatinine is a metabolite of creatine, and elevated serum creatinine levels are commonly used as a biomarker indicating impaired kidney function. Urinary creatinine, primarily filtered from the blood by the glomeruli and excreted in the urine, decreases in cases of kidney failure and increases with elevated blood creatinine levels. There is currently insufficient research on the direct link between urinary creatinine levels and the mechanisms of lung cancer development, and no direct studies on the relationship between urinary creatinine and lung cancer have been reported. Our research shows a causal relationship between elevated urinary creatinine and LSCC. We also found that SCLC and LSCC can cause elevated serum creatinine levels, similar to findings by Miroslava Sarlinova (38), who observed that both primary and secondary lung cancers cause significant increases in creatinine, glucose, citrate, and acetate, while pyruvate, lactate, alanine, tyrosine, and tryptophan significantly decrease. Elevated creatinine may result from obstructed creatine utilization and increased creatinine production, with kidney dysfunction also contributing.

Aminotransferases, including aspartate aminotransferase (AST) and alanine aminotransferase (ALT), are well-known biomarkers for liver damage. Studies have also linked elevated aminotransferases to systemic regulation of human diseases and metabolic functions (39). The AST/ALT ratio, also known as the De Ritis ratio, was initially proposed to study the etiology of hepatitis and is commonly used to distinguish between different causes of liver diseases such as fatty liver. Currently, the AST/ALT ratio is also employed as an effective biomarker for non-hepatic diseases like cardiovascular diseases, various cancers, and T2DM. Initially, a high AST/ALT ratio was reported to predict poor prognosis in non-metastatic renal cell carcinoma. Since then, further retrospective studies have validated the association between the AST/ALT ratio and cancer prognosis. However, no studies have specifically investigated its relationship with lung cancer. Our study indicates that the AST/ALT ratio is a risk factor for LADC and that SCLC can also cause an elevated AST/ALT ratio. Although direct studies on the relationship between the AST/ALT ratio and lung adenocarcinoma are lacking, some indirect evidence suggests a potential association. For instance, Wangyang Chen (40) examined the relationship between the AST/ALT ratio and various cancers, concluding that while there was no significant association with lung cancer overall, there was a notable connection with colorectal cancer risk. The primary mechanism suggested is that an elevated AST/ALT ratio may indicate liver damage, leading to the accumulation of toxic metabolites, which is associated with systemic inflammatory responses and metabolic diseases—all of which are factors related to colorectal cancer. This mechanism could similarly help explain a possible link between the AST/ALT ratio and lung adenocarcinoma, as lung adenocarcinoma may exhibit a stronger correlation with these factors compared to other types of lung cancer.Furthermore, Sofia Christakoudi (41) reported a negative correlation between ALT levels and lung cancer risk in men, suggesting that elevated ALT might be linked to obesity-related non-alcoholic fatty liver disease (NAFLD) and liver fibrosis, conditions that can reduce platelet counts and potentially lower lung cancer risk. This relationship may also partially explain why an elevated AST/ALT ratio might serve as a risk factor specifically for lung adenocarcinoma. As for the increase in the AST/ALT ratio often observed in patients with small cell lung cancer (SCLC), this may be due to the distinct characteristics of SCLC, which is generally more aggressive than other lung cancer types. SCLC has a higher propensity for liver metastasis, is associated with a more pronounced systemic inflammatory response, carries a higher risk of tumor lysis syndrome, and is more likely to produce ectopic hormone secretion. These factors collectively increase the risk of liver function impairment, and the AST/ALT ratio may serve as a marker for the extent of liver damage in this context.

Cystatin C (Cys-C) is a non-glycosylated, low molecular weight, basic protein composed of 120 amino acids (42). It is considered a housekeeping gene, with stable production by all nucleated human cells (40). Early studies indicated that cystatin C levels in healthy individuals were independent of age, muscle mass, or body mass index (BMI) (43, 44). Additionally, initial reports suggested that the production rate of cystatin C remains constant and is not altered under inflammatory conditions (45, 46). However, recent reports have found an association between serum cystatin C levels and inflammatory biomarkers such as C-reactive protein (CRP) (47, 48). Recent research has also linked serum cystatin C levels with tumors. Wojciech Naumnik (49) observed higher serum cystatin C concentrations in lung cancer patients compared to healthy individuals, a conclusion also reached by Qingyong Chen (50). Our study confirms these findings, showing that SCLC, NSCLC, and LSCC lead to elevated cystatin C levels. In cancer, increased cysteine protease activity, if not balanced by a corresponding increase in cysteine protease inhibitors, leads to the remodeling and degradation of extracellular matrix proteins—an event associated with tumor dissemination, invasion, and metastasis (51). Elevated expression of cystatins is expected to reduce tumor-related proteolytic activity, and indeed, evidence suggests that tumor-associated cystatins play an inhibitory role across various cancer types (52). Therefore, the high expression of cystatin C in lung cancer primarily acts as a tumor suppressor.

Glycated hemoglobin (HbA1c) is closely related to blood glucose levels and diabetes. The relationship between HbA1c and lung cancer is controversial. J C de Beer (53) found that elevated HbA1c does not lead to lung cancer. Similarly, Kai Liu (54), using Mendelian randomization analysis, concluded that HbA1c does not cause lung cancer, though he did not investigate whether lung cancer could cause elevated HbA1c levels. Our study also shows that HbA1c does not cause lung cancer, but SCLC and LSCC can lead to increased HbA1c levels. The elevation of glycated hemoglobin (HbA1c) in lung cancer is primarily attributed to cancer-induced hyperglycemia. The mechanisms by which lung cancer contributes to elevated blood glucose include: 1) Chemotherapy-Induced Pancreatic Damage: Certain chemotherapy agents can harm pancreatic islet cells, impairing insulin synthesis and secretion. Additionally, glucocorticoids, commonly used as adjuvants in lung cancer therapy, promote gluconeogenesis in the liver, inhibit glucose uptake and utilization in peripheral tissues, enhance the action of hyperglycemic hormones (such as growth hormone, epinephrine, and glucagon), and may also damage islet cell function. 2)Ectopic Hormone Secretion: Ejaz et al. (55) reviewed 43 cases of Cushing’s syndrome induced by ectopic ACTH secretion from tumors, finding that 48.9% of primary tumor sites were located in the chest. The most common symptoms were hyperglycemia (77%), venous thrombosis (14%), and infections (23%). The lung, as an endocrine organ, may contribute to hyperglycemia through the secretion of various bioactive substances. For instance, Unger et al. (56) detected glucagon in lung cancer tissue, which can promote hepatic glycogen breakdown and gluconeogenesis, thereby raising blood glucose levels. In some patients with elevated blood glucose, tumor treatment normalized glucose levels, only for hyperglycemia to return upon cancer relapse. 3)Cytokine-Mediated Insulin Resistance: Many advanced cancer patients exhibit low-grade CRP elevation, indicative of a chronic inflammatory state, which can raise IL-2, CRP, and cortisol levels, disrupting glucose metabolism. Additionally, tumor cells can secrete large amounts of IL-6 and TNF-α, leading to insulin resistance and subsequent hyperglycemia (57).

Our research indicates that SCLC and LSCC result in decreased eGFR. Cancer patients are at risk of acute kidney injury due to sepsis, direct damage to the kidneys from primary cancer, metabolic disorders, nephrotoxic effects of anticancer therapies, and hematopoietic stem cell transplantation (58–60). Nearly all hematologic and solid organ cancers are associated with tumor lysis syndrome, leading to uric acid nephropathy (61). Hypercalcemia occurs in up to 30% of patients with advanced cancer, often resulting in renal dysfunction due to AKI or CKD (61). Moreover, direct invasion and metastasis of cancer, infectious diseases caused by immunosuppression from cancer and its treatment, and various metabolic disorders can also lead to renal dysfunction (61, 62), contributing to decreased eGFR. Soonsu Shin (63) found that low eGFR is significantly associated with increased lung cancer risk, while Yutaka Hatakeyama (64) discovered that most cancers, particularly those of the kidney, urinary system, liver, or pancreas, cause decreased eGFR. Our study also found that SCLC and SCC result in decreased eGFR, but did not find that decreased eGFR causes lung cancer.

Gamma-glutamyltransferase (GGT) is located on cell membranes and is abundant in tissues with transport functions, such as the kidneys and biliary system (65). Elevated serum GGT is considered a marker of liver damage and alcohol consumption, but it is also a marker for various other diseases, including diabetes, cardiovascular diseases, and metabolic syndrome (66–68). Persistent elevation of serum GGT reflects chronic inflammation and oxidative stress, which contribute to tumor development and progression (69, 70). In lung cancer patients, alveolar macrophages and lymphocytes continuously produce GGT due to chronic inflammation-induced cytokines and growth factors (71, 72). Ye Jin Lee (73), through an observational study, linked persistently elevated GGT with lung cancer in men. D J F Brown (74) found significantly higher GGT levels in advanced lung cancer patients compared to healthy individuals. N V Liubimova (75) demonstrated that GGT is an effective marker for liver metastasis in lung cancer. However, Peter Groscurth (76) argued that lung cancer does not cause elevated GGT levels. Our study concludes that SCLC leads to decreased GGT levels. Our study found that small cell lung cancer (SCLC) may lead to decreased γ-glutamyl transferase (GGT) levels; however, the underlying mechanism has not yet been explored in the literature.

C-reactive protein (CRP) is a commonly used systemic marker primarily employed in diagnosing chronic and acute inflammation. It is produced by hepatocytes, and during an inflammatory response, such as infection or other injuries, certain molecular substances known as pro-inflammatory cytokines are generated. These cytokines stimulate hepatocytes to produce CRP. Therefore, elevated levels of CRP in the body may indicate an ongoing inflammatory response. In the context of cancer, CRP levels may also be elevated, making it a potential cancer biomarker. Jian Yin (77) studied the association between plasma high-sensitivity CRP (hsCRP) levels and lung cancer risk, finding no association between CRP levels and lung cancer risk in younger populations but a significant association in older populations. Elevated baseline CRP levels increased lung cancer risk in individuals with lower educational levels but not in those with higher educational levels. Stratification by BMI revealed a positive association between hsCRP levels and lung cancer risk in individuals with a BMI < 24, but no such association was observed in those with a BMI ≥ 28. Mengmeng Ji (78), through Mendelian randomization, demonstrated a significant correlation between circulating CRP levels and the risk of different histological subtypes of lung cancer. Although the etiological role of CRP in lung cancer has not been confirmed, circulating CRP may serve as an early diagnostic marker for lung cancer in current smokers. Our study did not find that CRP causes lung cancer, but it did show that non-small cell lung cancer (NSCLC) leads to elevated CRP levels. This is associated with the chronic inflammatory response induced by lung cancer. In the tumor microenvironment, large amounts of pro-inflammatory cytokines, such as interleukin-6 (IL-6) and tumor necrosis factor-α (TNF-α), are secreted. These cytokines stimulate the liver to synthesize and secrete C-reactive protein (CRP), resulting in increased serum CRP concentrations. Additionally, as the tumor grows, tissue necrosis may occur, releasing cellular contents and triggering further inflammatory responses that activate the immune system and stimulate additional CRP production.

Albumin is a water-soluble 65 kd protein synthesized by the liver. It is the most abundant blood protein in the human body, accounting for about half of the serum protein, and is responsible for the colloidal osmotic pressure of the blood. Some primary functions of albumin include binding insoluble molecules in the serum and transporting drugs and hormones (79). Hypoalbuminemia can result from malnutrition (insufficient intake), advanced liver disease (impaired synthesis), kidney disease (increased loss), and extreme catabolic states (increased breakdown) such as sepsis and metastatic cancer. Cancer can lead to hypoalbuminemia in several ways. Firstly, cancer patients may experience hypoalbuminemia due to the continuous consumption of nutrients, including albumin, by the tumor, leading to a deficiency of these essential nutrients in the body. Secondly, the metabolic products of the tumor may damage liver function, impairing the liver’s ability to synthesize albumin and thus causing a decrease in albumin levels. Additionally, cancer can cause cachexia, wasting, and reduced food intake, leading to insufficient nutrient intake and decreased albumin levels. Our study shows that NSCLC can lead to hypoalbuminemia.

Phosphate is crucial for normal cellular function as it provides fundamental components for DNA, cell structures, signal transduction, and energy production. Phosphate homeostasis is regulated by hormones such as fibroblast growth factor (FGF) (80) and parathyroid hormone (PTH). Hyperphosphatemia is usually caused by impaired kidney function. Conversely, hypophosphatemia may result from reduced dietary intake, malabsorption, or renal phosphate wasting due to genetic or acquired conditions. Phosphate toxicity is associated with tumorigenesis because high levels of inorganic phosphate in the tumor microenvironment can activate cell signaling pathways, promoting cancer cell growth. Ronald B. Brown (81) proposed that the association between alcohol and breast cancer is mediated by phosphate toxicity, i.e., the accumulation of excessive inorganic phosphate in body tissues. Phosphate homeostasis disruption leading to hypophosphatemia is common in cancer patients. Shreedhar Adhikari (82) elucidated the mechanisms and reasons for cancer-induced hypophosphatemia. Our study did not observe a causal relationship between phosphate and lung cancer, but it did find that NSCLC causes reduced phosphate levels.

Potassium is the most abundant electrolyte in active cells. Potassium homeostasis is maintained through various mechanisms, including internal and external processes. Urinary potassium generally reflects kidney function. Low urinary potassium excretion is associated with CKD progression. Kathrin Schilling (83) observed significantly lower urinary potassium levels in pancreatic cancer patients compared to healthy controls. Our study shows that NSCLC causes decreased urinary potassium levels.

Serum urea is the end product of protein metabolism, filtered through the renal glomeruli, and excreted from the body. There are very few studies evaluating the impact of serum urea on cancer development.Yandi Sun (84), through Mendelian randomization, demonstrated a positive association between serum urea levels and female-specific RCC (renal cell carcinoma) risk. Haoyan Chen (85), through single-cell transcriptomics, microbiome analysis, metabolomics, and clinical analysis of colorectal adenomas and cancer tissues, found significant activation of host urea cycle metabolism during colorectal cancer development, with low bacterial urease abundance and high urea load detected in colorectal cancer. M. C. Winter (86) proved that elevated pre-treatment serum urea is an important predictor of early mortality in SCLC. Our study shows that LSCC increases serum urea levels. Our study indicates that squamous cell carcinoma may elevate serum urea levels, potentially due to tumor-induced kidney dysfunction and the high catabolic state associated with malignancy.

SHBG is a glycoprotein that binds with high affinity to 17β-hydroxy steroid hormones, including testosterone and estradiol. Its concentration regulates the balance between bound and free hormones, serving as a transport carrier and modulating the bioactivity of sex hormones (87). Women have SHBG levels twice as high as men, reducing exposure to androgens and estrogens (88). Researchers have found associations between sex hormones and cancer in several studies. Zoë Hyde and her team discovered that higher testosterone levels are associated with an increased risk of prostate and lung cancer (89). Niki Dimou found that SHBG levels are negatively correlated with breast cancer risk (90). Katherine Ruth observed a positive correlation between female testosterone levels and endometrial cancer risk (91). Furthermore, according to the Women’s Health Initiative, individuals with the highest SHBG levels are more than twice as likely to develop colon cancer compared to those with the lowest SHBG levels (92). Our study results suggest a bidirectional causal relationship between squamous cell carcinoma and SHBG.

Alkaline phosphatase (ALP) is a glycoprotein that catalyzes hydrolysis and phosphate transfer reactions. Elevated serum ALP has been reported in bone and liver-related diseases (93, 94). Additionally, serum ALP has been found to be an independent prognostic factor in NSCLC, gastric cancer, breast cancer, and other cancer types. Elevated ALP levels are also associated with bone or liver metastases in patients with lung cancer, prostate cancer, breast cancer, and other types of cancer (95). TAO YANG (95) found that elevated serum ALP levels in NSCLC patients are associated with bone or liver metastases. N Walach (96) found that compared to CEA levels, LAP scores are a more reliable marker for detecting lung cancer, especially metastatic lung cancer. Our study similarly found that adenocarcinoma causes elevated LAP, consistent with the aforementioned studies.

In this study, we conducted a comprehensive assessment of the association between blood/urine biomarkers and lung cancer using a bidirectional Mendelian randomization approach. Our findings identified several potential causal relationships between blood and urine biomarkers and lung cancer, with some confirmed through FDR-adjusted significance. This research can contribute to understanding the mechanisms underlying lung cancer development. Additionally, we suggest that screening for these blood and urine biomarkers can help identify individuals with abnormalities who may benefit from LDCT screening, enabling early clinical intervention, regardless of age or smoking status.

Our study comprehensively investigated the associations between blood/urine biomarkers and lung cancer. The sample size maximized the power of the genetic analysis. However, this study has several limitations. First, the lung cancer dataset is from the Finnish database, which is predominantly composed of individuals of European descent. Therefore, caution is needed when applying our findings to other ethnic populations. Second, the use of residuals might affect the magnitude of the reported effects; future studies should consider alternative methods to further validate our findings. In future research, it is necessary to use independent clinical samples or cohorts to validate our findings and to investigate the potential biological mechanisms underlying the associations between candidate blood and urine biomarkers and lung cancer. Third, the methods and accuracy of biomarker measurements in the UK Biobank could influence our results. Fourth, some results exhibit heterogeneity, and although we used the IVW random-effects model and multiplicative effects model to verify consistency, this does not entirely resolve the issue of heterogeneity. Fifth, we applied the modified FDR correction for p-values, which may impact the results.

In our study, while we used bidirectional two-sample Mendelian randomization to assess the potential causal relationships between blood and urinary biomarkers and lung cancer, we acknowledge that more sophisticated analyses are needed to further elucidate the connections between these biomarkers and lung cancer development. In particular, the scPagwas method developed by Ma et al. (2023) offers an innovative approach that combines single-cell RNA sequencing (scRNA-seq) data with summary statistics from genome-wide association studies (GWAS), identifying cell subpopulations and pathways associated with complex diseases (97). Applying this method may help uncover lung cancer-related immune cell types and key proteins that mediate the causal relationships between blood and urinary biomarkers and lung cancer.

Moreover, Ma et al. (2022) demonstrated the potential of integrating blood cell scRNA-seq data with GWAS data in identifying risk genes, inflammatory factors, and immune cell types associated with severe COVID-19. This work provides a valuable framework for investigating potential links between blood and urinary biomarkers and lung cancer (98). These findings underscore the importance of single-cell analyses in identifying disease-related cellular subpopulations and may offer crucial insights into the pathophysiology of lung cancer.

Although our study did not directly employ the scPagwas method, these studies highlight the promise of adopting similar approaches in future research to identify specific immune cell subpopulations and key proteins involved in the initiation and progression of lung cancer. Such approaches could deepen our understanding of the causal relationships between blood biomarkers and lung cancer and may help identify novel therapeutic targets. Future studies could utilize scPagwas to integrate lung cancer GWAS data with blood cell scRNA-seq data to identify lung cancer-associated cell subtypes, providing a stronger theoretical basis for early screening and therapeutic monitoring of lung cancer.

## Data Availability

The original contributions presented in the study are included in the article/**Supplementary Material**. Further inquiries can be directed to the corresponding author.
