# Supplementary figures and images for: Mendelian randomization study of the relationship between blood and urine biomarkers and lung cancer
Source: Front Oncol. 2024 Dec 2;14:1453246. doi: 10.3389/fonc.2024.1453246 (PMC11646849; doi:10.3389/fonc.2024.1453246)

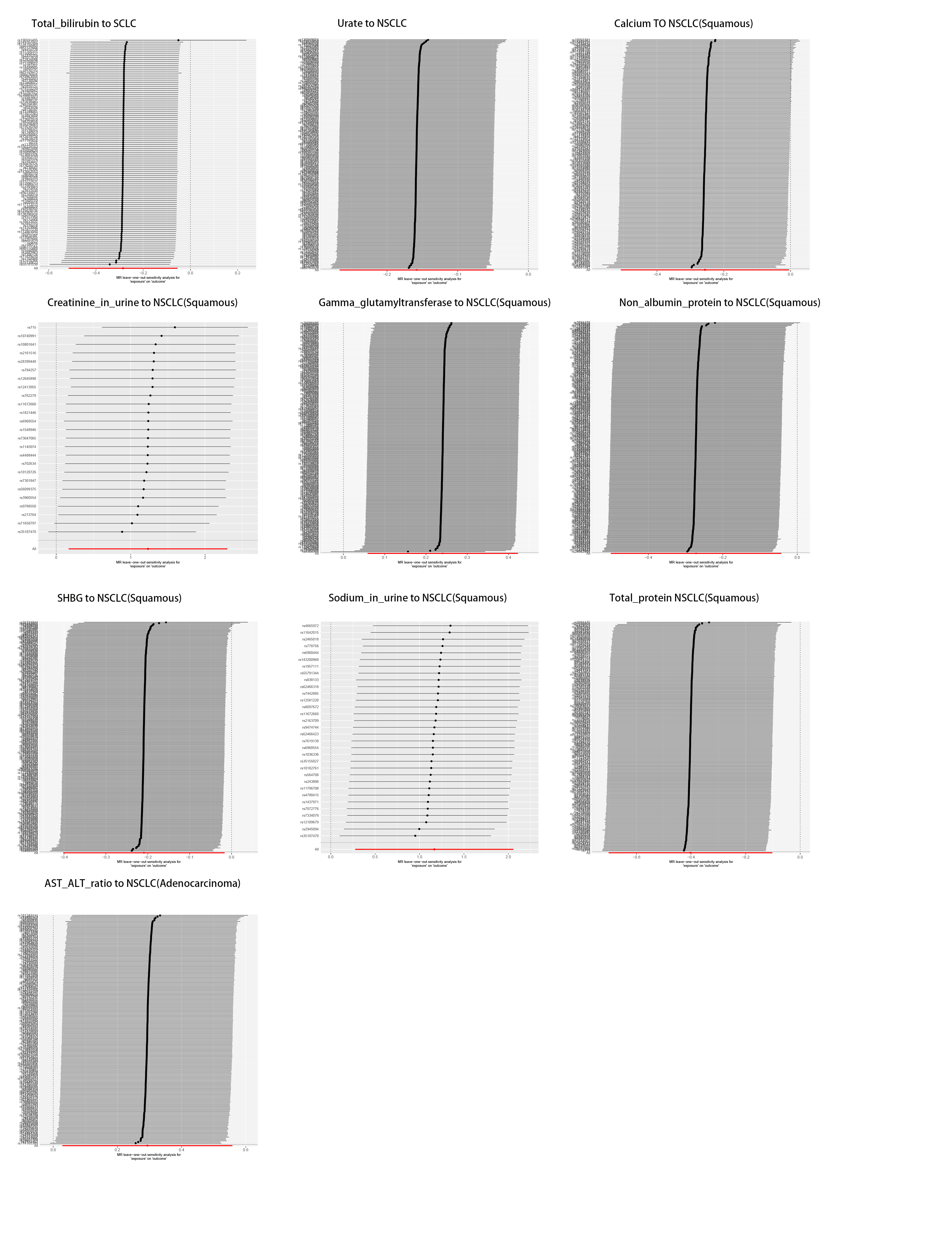

Supplement: Supplementary file 1 [file DataSheet1.zip › supplementary/S1.tif]

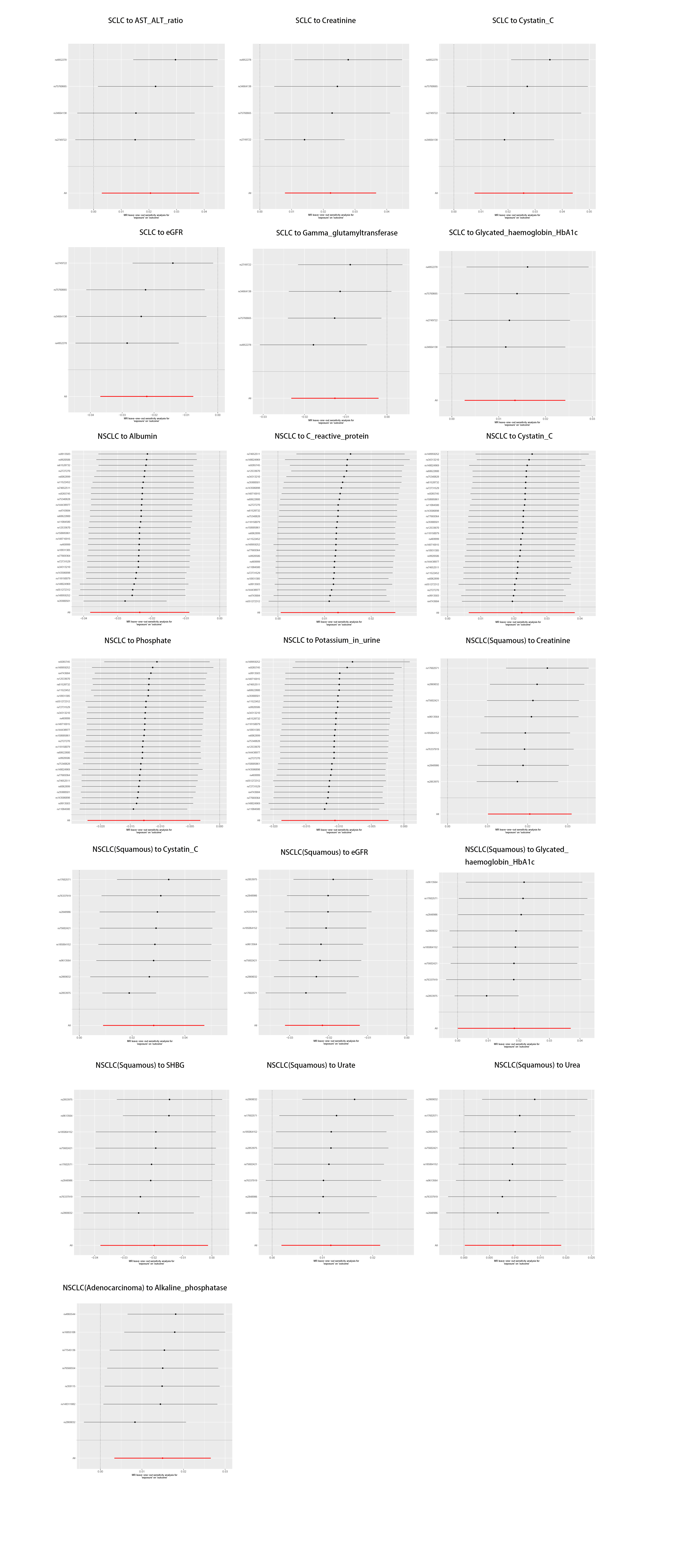

Supplement: Supplementary file 1 [file DataSheet1.zip › supplementary/S2.tif]
